# Continuing Professional Development—Radiation Therapy

**DOI:** 10.1002/jmrs.70016

**Published:** 2025-08-14

Maximise your continuing professional development (CPD) by reading the following selected article and answering the five questions. Please remember to self‐claim your CPD and retain your supporting evidence. Answers will be available via the QR code and published in JMRS—Volume 72, Issue 4, December 2025.

## Proton Therapy Patient Selection Methods and the Impact of COVID‐19: A Cross‐Sectional International Survey

Lucy Wood, Eileen Giles, Lisa Cunningham, Hien Le, Nicole Zientara, Michala Short, https://doi.org/10.1002/jmrs.885.

1. Why is patient selection an important consideration for proton therapy?
  - All patients are required to travel abroad for treatment
  - Proton therapy is generally less effective than traditional photon therapy
  - The treatment technology is still regarded as experimental
  - Proton therapy facilities are limited in availability, and the treatment is expensive
2. What has been the most used patient selection method for proton therapy over the past 5 years?
  - Insurance‐based criteria
  - Diagnosis or indication‐based lists
  - Model‐driven selection
  - Comparative planning for target coverage and tumour control probabilities
3. How did most proton therapy centres adjust their patient selection methods during the COVID‐19 pandemic?
  - Most centres limited treatment to only paediatric patients
  - Most centres prioritised only emergency cases for proton therapy
  - Most centres continued using their pre‐pandemic selection methods
  - Most centres implemented temporary selection criteria prioritising only high‐risk cancer types
4. How did some proton therapy centres modify treatment schedules for COVID‐19 positive patients?
  - They delayed treatment until the patient tested negative
  - They scheduled treatments at specific times and enhanced safety protocols
  - They permanently discontinued treatment
  - They referred all patients to conventional radiotherapy
5. Which patient group was more likely to experience delays or suspensions in proton therapy treatment during the COVID‐19 pandemic?
  - Patients with high‐risk cancers
  - International emergency cases
  - Patients with low‐risk or benign tumours
  - Many paediatric patients

## Answers

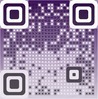

Scan this QR code to find the answers.
